# Applied systems thinking: unlocking theory, evidence and practice for health policy and systems research

**DOI:** 10.1093/heapol/czab062

**Published:** 2021-06-23

**Authors:** Aku Kwamie, Solip Ha, Abdul Ghaffar

**Affiliations:** Alliance for Health Policy and Systems Research, World Health Organisation, Avenue Appia 20, 1211 Geneva, Switzerland; Alliance for Health Policy and Systems Research, World Health Organisation, Avenue Appia 20, 1211 Geneva, Switzerland; Alliance for Health Policy and Systems Research, World Health Organisation, Avenue Appia 20, 1211 Geneva, Switzerland

**Keywords:** Health systems, health systems research, policy processes

## Abstract

While systems thinking has been generally acknowledged as important to the field of health policy and systems research (HPSR), it remains underutilized. In particular, systems thinking has been perceived as predominantly conceptual, with fewer applications of systems thinking documented. This commentary makes three key points, namely that (1) advances in applied systems thinking in HPSR have been hindered by an imprecision in terminology, conflating ‘[health] systems approaches’ with complex adaptive systems theory; (2) limited examples of applied systems thinking have been highlighted and recognized in research, but have not been fully and equally appreciated in policymaking and practice and (3) explicit use of theory, long-term research-policy collaborations and better documentation of evidence can increase the use and usefulness of applied systems thinking in HPSR. By addressing these matters, the potentials of systems thinking in HPSR can be truly unlocked.

**Key messages**

- Advances in applied systems thinking in health policy and systems research have been hindered by an imprecision in terminology.
- Limited examples of applied systems thinking have been highlighted and recognized in research but have not been fully and equally appreciated in policymaking and practice.
- Explicit use of theory, long-term research-policy collaborations and better documentation of evidence can increase the use and usefulness of applied systems thinking in HPSR.

## Introduction

For more than a decade, systems thinking has been accepted as integral to health policy and systems research (HPSR). Since the publication of the Alliance flagship report, *Systems Thinking for Health Systems Strengthening* (De Savigny and Adam, 2009), there has been significant growth in the literature on systems thinking in public health (Chughtai and Blanchet, 2017). However, nearly half of these peer-reviewed articles have been calls for more systems thinking approaches, with fewer empirical examples of applied systems thinking (Carey *et al.*, 2015). Despite the important body of knowledge that has formed through the articulation of systems thinking frameworks (Adam and De Savigny, 2012), collection of case studies (Adam, 2014), and explanations of tools and methods (De Savigny *et al.*, 2017), systems thinking *in practice*, particularly in low- and middle-income countries (LMICs), has remained broadly overlooked as a means of addressing health system challenges. The COVID-19 pandemic has only underscored the systemic difficulties that arise when health actions are considered in discrete and short-term fashion, linkages to broader social systems are not fully considered and potential consequences are not holistically thought through.

This commentary explores reasons for the continued underutilization of applied systems thinking and offers two possibilities. First, we posit that systems thinking in HPSR has been constrained by an imprecision in terminology, conflating language between approaches to health systems strengthening and complex adaptive systems (CAS) theory. Second, we reflect on the fact that systems thinking has remained primarily the domain of researchers, with little to no uptake in policymaking and practice. We conclude by outlining a way forward to refine the science and policy readiness of systems thinking to address both the enduring and emerging challenges that face health systems today.

## What do we mean by ‘systems approaches’?

The dominant framework to shape health systems strengthening efforts has been the WHO Health Systems Framework, which articulates six ‘building blocks’ as a way to communicate the functional domains that make up health systems, and where multiple effects of health interventions are observed beyond disease- or programme-specific perspectives (World Health Organisation, 2007). The 2009 Alliance report built on this framework, focusing on using systems thinking to design and evaluate health interventions (De Savigny and Adam, 2009). Since then, approaches variously described as ‘systemic’, ‘system-based’, ‘system-wide’ and ‘system-level’ have been used as intervention entry-points to reflect this broader set of interactions. Tangible shifts towards systems approaches for health interventions have been observed in greater appreciation of context specificity, broader stakeholder engagement and more coordinated work across multiple building blocks.

This, however, is distinct from CAS *theory* that examines the nature of the interactions themselves that occur between elements of a system, and how these interactions display adaptive features such as holism, non-linearity, feedback, self-organization, delays and emergence (Holland, 2006). Systems thinking, which stems from complexity theory (itself longstanding and evolved into several scientific streams), analyses these interactions between systems’ components to explain how and why they give rise to observed system outcomes and behaviours. While health systems are acknowledged to be CASs, CAS theory has been little applied in their analysis (Paina and Peters, 2011). In reality, terminology around ‘[health] systems approaches’ has been used interchangeably with systems thinking. The result has been a general consensus on the importance and need of systems thinking in health systems strengthening, with limited theorizing and evidence of how to engage with systems thinking in practice.

## Systems thinking in and for policymaking and practice?

Additionally, systems thinking has often been considered too conceptual to be policy relevant. Broader policy environments that exhibit bureaucratic administrative structures and cultures of government that incentivize linearity and efficiency, privilege standardization and uniformity, presume command-and-control, trail historical legacies and avoid risk (Chapman, 2004) have tended to be resistant to the diffusion of systems thinking. In many LMICs, resource dependencies and constraints further magnify rigid hierarchical authority that hampers systems thinking capabilities. This has meant that often, where systems thinking has been introduced—usually in short-term, tool-based interventions—such efforts are valued but prove difficult to institutionalize (Kwamie *et al.*, 2014). Exposure to systems thinking concepts alone is insufficient to engender systemic change.

More recently, examples from high-income countries (HICs) advocate governments making significant changes in order to facilitate greater systems thinking in their workings, including promoting adaptive forms of leadership and orienting towards more systemic (rather than output-based) evaluations (OECD, 2017). An illustration from Australia (Haynes *et al.*, 2020) typifies the concrete impacts on policy design, policy narratives and implementation that can be generated when policymakers actively engage with systems thinking. However, such efforts are successful and sustained due to significant resource intensity in terms of people, money and time. While such examples are encouraging, they do raise questions about practicability in settings where such resources are under pressure.

## The way forward: what is needed?

Systems thinking in HPSR—as both a lens and a set of tools and methods—needs reinvigoration. Our recent explorations for LMIC examples of applied systems thinking in policymaking and practice through the literature and consultation with various experts returned very little. While this does not mean that systems thinking is not being practiced—it may be, but not being called such, or not being published in either peer or grey literature—it does imply that there is need to increase the opportunities for applied systems thinking. We offer the following suggestions.

First, applied systems thinking in HPSR should be more explicitly theorized. This will help to clarify the issues of terminology. Implementation of policy and programme interventions can more fully use CAS theory in their analyses, either prospectively to build understanding of how interventions might interact with their contexts to bring about their expected (and other) outcomes or retrospectively to understand, based on the observed outcomes, what really happened. Various systems thinking tools, such as systems modelling, process mapping, causal loop diagrams and social network analysis can be usefully deployed in this regard.

Second, greater government investment and institutional incentives that foster long-term collaboration and strengthen science-policy partnerships should be pursued in order to augment the capacities and practice of systems thinking in policymaking.

Third, and relatedly, evolutions in funding norms should be interrogated to better enable systems thinking in LMICs. Increasingly, funders and charities in HIC social sectors are recognizing the restrictions posed by small scale and fragmented funding; some are experimenting with pooling resources through longer term funder partnerships to be able to address complexity and adaptive approaches (Abercrombie *et al.*, 2015; Co-Impact, 2019). These are compelling ideas that have yet to (and should) gain traction amongst international funders.

Fourth, greater documentation on applied systems thinking is needed to build a strong evidence-base of how applied systems thinking is done in practice, what it costs (to engage and not engage with it) and how its outcomes differ as a result. Indeed, the fact that experiences of applied systems thinking are not well-reflected in the literature is a limitation to this commentary.

What could applied systems thinking in LMICs look like in practice? Policymakers and practitioners grounding their systems questions in their contexts, seeking out researchers to accompany them with theories, tools and methods able to address them, and enabled by funding approaches that support networked institutions to document and share their learnings and failures over the long term. In this way, renewed interest in applied systems thinking in HPSR, supported by explicit theory, enhanced capacities and improved evidence, can catalyse the use and utility of systems thinking for strengthening health systems
